# An Italian Neurorehabilitation Hospital Facing the SARS-CoV-2 Pandemic: Data From 1207 Patients and Workers

**DOI:** 10.3389/fneur.2020.584317

**Published:** 2020-10-09

**Authors:** Antonino Salvia, Giovanni Morone, Marco Iosa, Maria Pia Balice, Stefano Paolucci, Maria Grazia Grasso, Marco Traballesi, Ugo Nocentini, Rita Formisano, Marco Molinari, Angelo Rossini, Carlo Caltagirone

**Affiliations:** Fondazione Santa Lucia, IRCCS, Rome, Italy

**Keywords:** coronavirus, COVID-19, rehabilitation, molecular test, swab, SARS-CoV-2, hospital design and construction

## Abstract

**Objective:** The aim of the present observational study is to report on the data from a large sample of inpatients, clinical staff and other workers at an Italian neurorehabilitation hospital dealing with SARS-CoV-2 infections, in order to analyze how it might have affected the management and the effectiveness of neurorehabilitation.

**Methods:** The data on infection monitoring, obtained by 2,192 swabs, were reported and compared among 253 patients, 722 clinical professionals and 232 other hospital workers. The number of admissions and neurorehabilitation sessions performed in the period from March-May 2020 was compared with those of the same period in 2019.

**Results:** Four patients and three clinical professionals were positive for COVID-19 infection. Six out of these seven people were from the same ward. Several measures were taken to handle the infection, putting in place many restrictions, with a significant reduction in new admissions to the hospital (*p* < 0.001). However, neither the amount of neurorehabilitation for inpatients (*p* = 0.681) nor the effectiveness of treatments (*p* = 0.464) were reduced when compared to the data from 2019.

**Conclusions:** Our data show that the number of infections was contained in our hospital, probably thanks to the protocols adopted for reducing contagion and the environmental features of our wards. This allowed inpatients to continue to safely spend more than 3 hours per day in neurorehabilitation, effectively improving their independence in the activities of daily living.

## Introduction

The World Health Organization (WHO) characterized the novel severe acute respiratory syndrome coronavirus-2 (SARS-CoV-2) outbreak as a pandemic on March 11th 2020 (1). In response to this, many governments implemented a series of emergency containment measures, including social restrictions and the quarantine of positive and suspect cases. Italy was the first Western country with a wide diffusion of coronavirus disease (COVID-19), so it could be important for other countries to analyze in depth the Italian case-study (2). In April, Iosa and coworkers suggested a dynamic analysis of the Italian case-fatality ratio to gain deeper insight into the course of the COVID-19 pandemic, and we anticipated the need to prepare rehabilitation units. Both people with COVID-19 sequelae related to motor and respiratory functions and patients with neurological disorders (3), not infected but needing neurorehabilitation in the time of this pandemic, could not wait to be treated (4–6). Several measures, including those imposed by the Decrees of the Italian President of the Council of Ministers, were taken to tackle the infection in rehabilitation hospitals with the aim of monitoring the insurgence of epidemic outbreaks (4). As nosocomial transmission is a severe problem in relation to the condition of inpatients, any action should be taken to minimize the risk of transmission among patients and clinical staff (7). However, an analysis of the impact of these measures is lacking in the literature. A recent study analyzed the outcomes of outpatients with pre-existing neurological disorders reporting the prevalence of symptoms of COVID-19 infection (8). The authors found that the presence of neurological chronic diseases did not increase the prevalence of COVID-19 infection, but the burden of neurological disorders was worsened by the lockdown. These problems could be even more dramatic for inpatients. Indeed, hospital access to people with neurological impairments due to brain or spinal cord injury in the subacute phase was often postponed as a consequence of infection. However, the neurorehabilitation of these patients is time-dependent and cannot be delayed, nor can it be reduced in intensity (9, 10). Furthermore, in Italy, there was an increased pressure on high specialty neurorehabilitation wards to admit patients with severe neurological disabilities (sometimes even with unstable vital functions and a high risk of medical complications) due to the sudden need for intensive care units to free up beds for COVID-19 positive patients with respiratory insufficiency. Other countries are now facing the same problem. In this scenario, there is a need for a quantitative analysis of the impact of the adopted restriction measures on inpatients needing neurorehabilitation, especially in hospitals exposed to the risk of COVID-19 infection.

The aim of the present observational study is to report on the data regarding COVID-19 infection monitoring in a large sample of inpatients needing neurorehabilitation, clinical staff and other hospital workers, analyzing how it might have affected the management and the efficacy of neurorehabilitation.

## Materials and Methods

This was an observational study performed on data collected during the monitoring of hospitalized inpatients and people working in our hospital from April 30th to May 26th 2020. People were classified into three categories: inpatients, clinical staff (medical doctors, psychologists, nurses, therapists, health care assistants, etc.) and other hospital workers (administrative staff, cleaners, etc.). They underwent repetitive series of swabs related to the program monitoring at out hospital during the COVID-19 pandemic.

### Protocols

**Figure 1:**
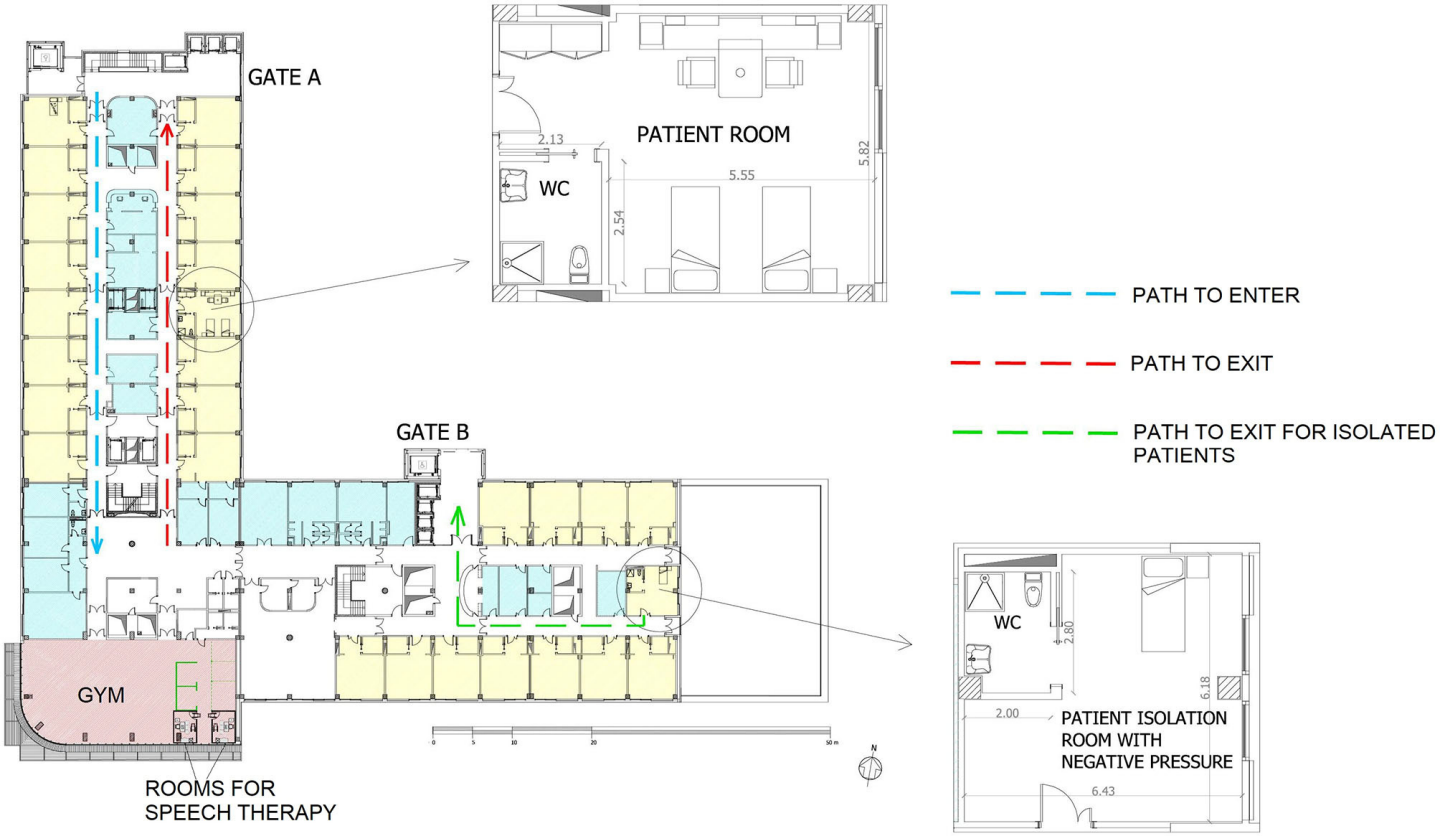
The planimetry of one of the six identical wards of our hospital. In details are also shown the planimetry of one of the twenty-seven standard patient's rooms of the ward (in yellow) and the special isolation room with negative pressure. In blue the rooms of doctors and in pink the gym. Stairs and elevators are located in proximity to each gate. The dotted rows show the pathway defined for human traffic during the analyzed period: the blue pathway is that for entering into the ward, the red one for exit from the ward, and the green one that related to a patient isolated in the special room. All the measures are expressed in meters.

During the SARS-CoV-2 pandemic, the Italian Society of Neurological Rehabilitation indications were followed for the reorganization of neuro-rehabilitation activities (4). Moreover, we also changed our protocols in the following aspects:

- Our neurorehabilitation units admitted patients with subacute neurological impairments due to severe acquired brain lesions or stroke coming from an acute unit only if they were not affected or suspected to be infected with COVID-19 (with two negative nasal swabs performed within the last 48 h before discharge from acute wards).
- Our hospital is formed by six identical wards for inpatients, with 26 bedrooms having only two beds each (each room was 46 square meters with a bathroom inside), according to the requirements defined by Italian law, plus one room with negative pressure, and the availability of a gym in each ward (as shown in Figure 1). We planned three different pathways for human traffic within each ward: one for entering, one for exit, and one specific for isolated patient with different elevators just used for them, as shown in Figure 1. This figure also shows that each ward has a gym in which there were two rooms for speech therapy. The detail of one bed-room and of the isolation room with negative pressure were also shown in that figure.
- Healthy people, such as workers and allowed visitors, could enter into the hospital only if their body temperature was lower than 37.5°C (if higher, home isolation was suggested). Employees commute from home, no specific restrictions to social activities were required by the hospital direction, but they were required to strictly follow the restrictions of the lockdown programmed by Italian government for all the citizens (for example, in the study period, in Italy most of the shops were closed, restaurants were closed, religious meetings were forbidden).
- Posters with behavioral/health general rules (such as about social distancing in rooms, maximum number of people allowed into elevators at the same time, hand hygiene recommendations, use of protective devices, and so on) were positioned at each entrance and within all the complex operative units and the number of hand-sanitizing gel dispensers in the whole hospital was increased.
- A specific guideline has been drawn up describing prevention and control measures during the management of suspected and confirmed cases of COVID-19 in our facility, and several training sessions have been held with the health care professionals for the correct use of SARS-CoV-2 personal protective equipment (PPE) such as surgical masks, whereas suspected cases were cared by clinical staff using medical cap, eye-visor, face shield, N95 respiratory mask, double disposable gloves, medical protecting coverall, leg cover waterproof boots.
- Reorganization of work shifts (medical and non-medical staff) with a reduction in some activities in order to reduce contact and movements (for example, encouraging staff to work from home for administrative activities).
- After the first case of a positive swab in our hospital (April 30th), neither new admissions (up to May 21st) nor visitors (for the entire month of May and the first weeks of June, with only a few exceptions for caregivers staying in the same room of patients needing continuous assistance after two consecutive negative swabs) were allowed. An extraordinary plan for daily cleaning and sanitization of the wards was putted in action.
- Outpatient activities and/or day hospital rehabilitation were reduced (only activities that cannot be postponed according to regional guidelines were maintained in different areas and different clinical staff from those of inpatients to avoid contacts between these two populations).
- Strengthening of patient and caregiver support networks, also through information technologies and an emphasis on the role of tele-rehabilitation.
- Seven sessions of swab analyses for monitoring patients and workers were carried out from the end of April to the end of May.

### Epidemiological Analysis

From 30th April, we started an epidemiological analysis on the inpatients and employers of our hospital with the main aim to identify asymptomatic subjects. The whole sample was formed of 1,207 people: 253 inpatients, 722 clinical professionals and 232 other hospital workers. Among the patients, 153 were affected by stroke, 54 by traumatic brain injury, 31 by spinal cord injury, 9 by multiple sclerosis, 2 by Parkinson's disease, and 4 by orthopedic problems. Our hospital provides six wards for inpatients. Workers and patients were assigned to the same ward for the entire investigation period, in order to limit transfers.

### Data Analysis

Data are reported in terms of mean and standard deviation for continuous measures, absolute frequency for counted discrete measures, median and interquartile range (IQR = third–first quartiles) for ordinal measures of clinical scales and not normally distributed variables. Odds ratios were computed together with relevant 95% confidence intervals (95% CI) and inferred by the chi-squared test. Paired *t*-tests were performed to compare the data related to March, April and May 2020 and 2019 in terms of time spent in neurorehabilitation activities. Mann–Whitney *U*-tests were performed for comparing the Barthel Index (BI) score at admission and discharge, effectiveness and length of stay in the period from March to May 2020 vs. the same period in 2019. The effectiveness was computed as the percentage improvement obtained in terms of the BI-score with respect to the maximum achievable one. The level of statistical significance was set at 5%.

## Results

### Epidemiological Data of Swab Analysis

During the study period, the statistics of the outbreak in Italy reported 104,664 new contagions in March, 99,671 in April, and 27,534 in May. In Lazio (the Italian region of our hospital and in which resided our employees) the number of contagions were 3,092 in March, 3,521 in April and 1,112 in May, on a population of about 5.9 million of people (11).

In our hospital, a total of 2,192 nasal swabs were performed in <1 month: 13 swabs on April 30th finding one nurse positive; 62 swabs on May 2nd finding another nurse and two stroke patients positive (these patients came from the same ward as the first positive case); 1,093 swabs on May 4th and 5th with positive findings in other two stroke patients (from the same ward) and one therapist (from another ward); no more positive cases were found in the subsequent analysis of 31 swabs on May 7th, 151 swabs between 11th and 12th, 738 between 18th and 19th, 104 swabs on 26th May. All seven positive cases (corresponding to a prevalence of 0.6%) were asymptomatic. Familiars were advised. According to the regional guidelines, positive patients were immediately isolated in the special room with negative pressure and transferred within 6 h to a regional dedicated Covid-hospital (during this short time, the patients did not receive any rehabilitation session). After the recovery in that hospital and the following negativization of two consecutive swabs, two out of the four patients were discharged at home and the other two were re-admitted to our hospital to continue the neurorehabilitation. The cases of positive employers were notified to the competent authorities, and these subjects were quarantined at home for 14 days in charge to family physician, and they were allowed to return to work after 2 negative nasal swabs performed within the last 48 h.

The cumulative data are reported in Table 1. The odds ratio of being infected by SARS-CoV2 was not statistically different between patients and clinical staff, although it was close to the significance threshold (OR = 3.85; 95% CI: 0.86–17.3; *p* = 0.0588). Conversely, an odds ratio of 64.8 (95% CI: 7.7–545; *p* < 0.001) was found in favor of being hospitalized or working in the same ward. For a comparison with the data of the outbreak during the study period, in Italy there were 104,664 new contagions in March, 99,671 in April and 27,534 in May. In Lazio (the Italian region of our hospital and in which resided our employees) the number of contagions were 3,092 in March, 3,521 in April and 1,112 in May.

**Table 1:** Data on subjects classified as patients, clinical staff or other hospital workers.

**Categories of people**	**Age (years old)**	**Sample *N***	**Gender (female %)**	**Number of tests**	**Positive swab**
**Patients**	66.3 ± 16.2	253	35%	560	4
**Clinical staff**	44.0 ± 11.8	722	64%	1,361	3
**Other workers**	47.6 ± 12.4	232	59%	271	0
**Total**	50.7 ± 16.4	1207	57%	2,192	7

### Number of Treated Patients

**Figure 2:**
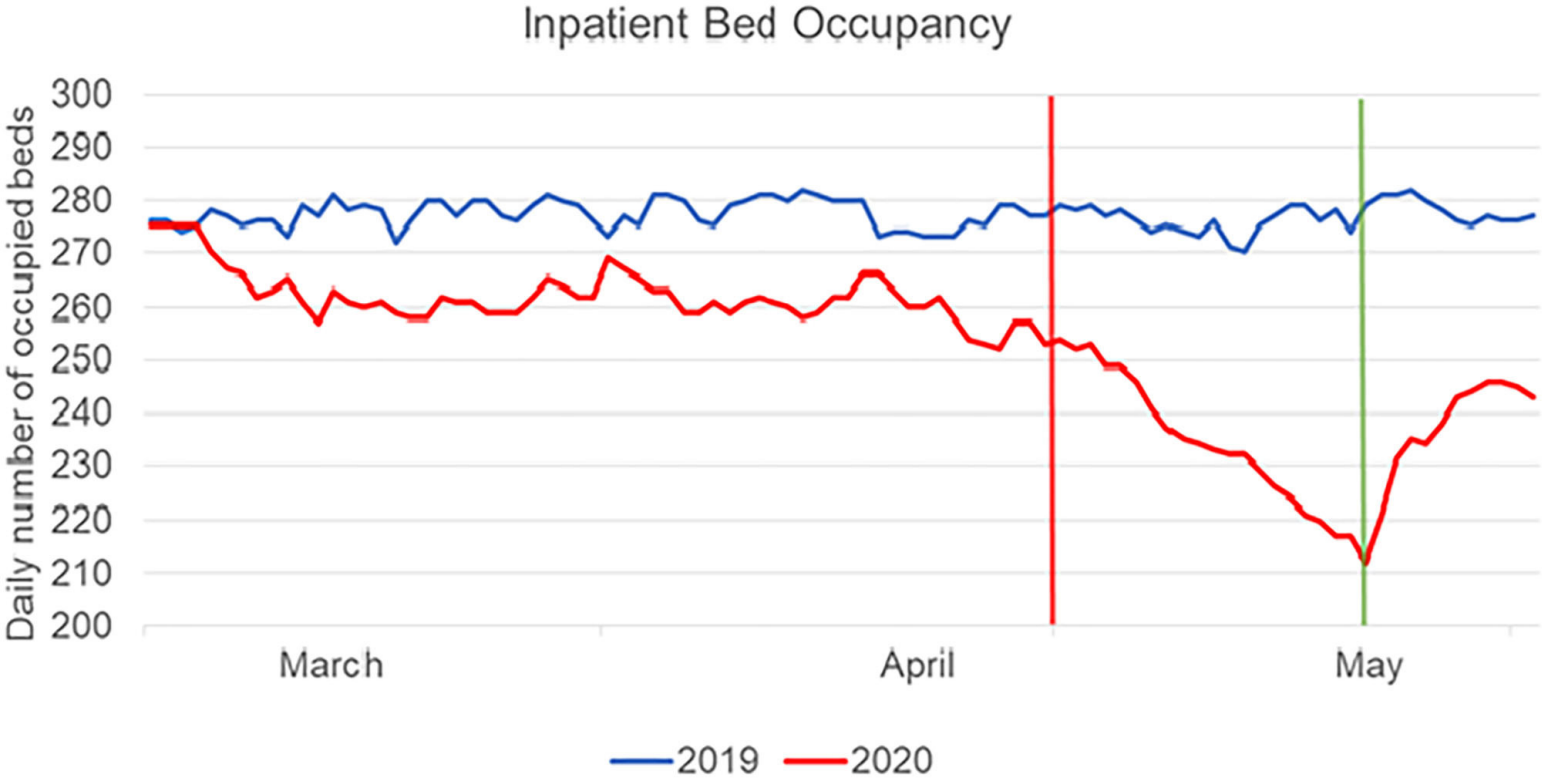
Hospital bed occupancy in 2019 (blue line) and in 2020 (red line) for the period from March to May. The vertical red line represents the day of the first positive case in our hospital, corresponding to the beginning of the temporary halt to new admissions. The green line represents the last day of this period of halted admissions.

The admission of patients to the hospital was stopped immediately after the first positive swab. This measure implies a reduction in inpatients, as shown in Figure 2. During the period from March to May 2020, 89 patients were admitted (median BI-score = 12, IQR = 26). In the same period of 2019 the number of admissions were 180; however, the median BI-score was not significantly different (BI-score = 13, IQR = 30, *p* = 0.468).

The hospital bed occupancy was significantly different in the entire period from March to May 2020 with respect to the same period in 2019 (*p* < 0.001), especially after the first positive case, when the temporary halt to new admissions occurred, as shown in Figure 2. The rehabilitation of outpatients (day hospital) was even more significantly decreased: it was 36% of planned occupancy in March, 3.8% in April and 8.6% in May, whereas the matching percentages in 2019 were 106, 102.1, and 103.6%, respectively (*p* = 0.010).

From an economic point of view, the amount of loss of income related to the reduction of Day Hospital was 64% in March, 96% in April, and 92% in May; whereas that of inpatients was 5, 6, 15%, respectively. The loss related to other outpatient hospital services were 73, 81, and 61%, respectively.

### Number of Treatments and Their Effectiveness in Inpatients

**Figure 3:**
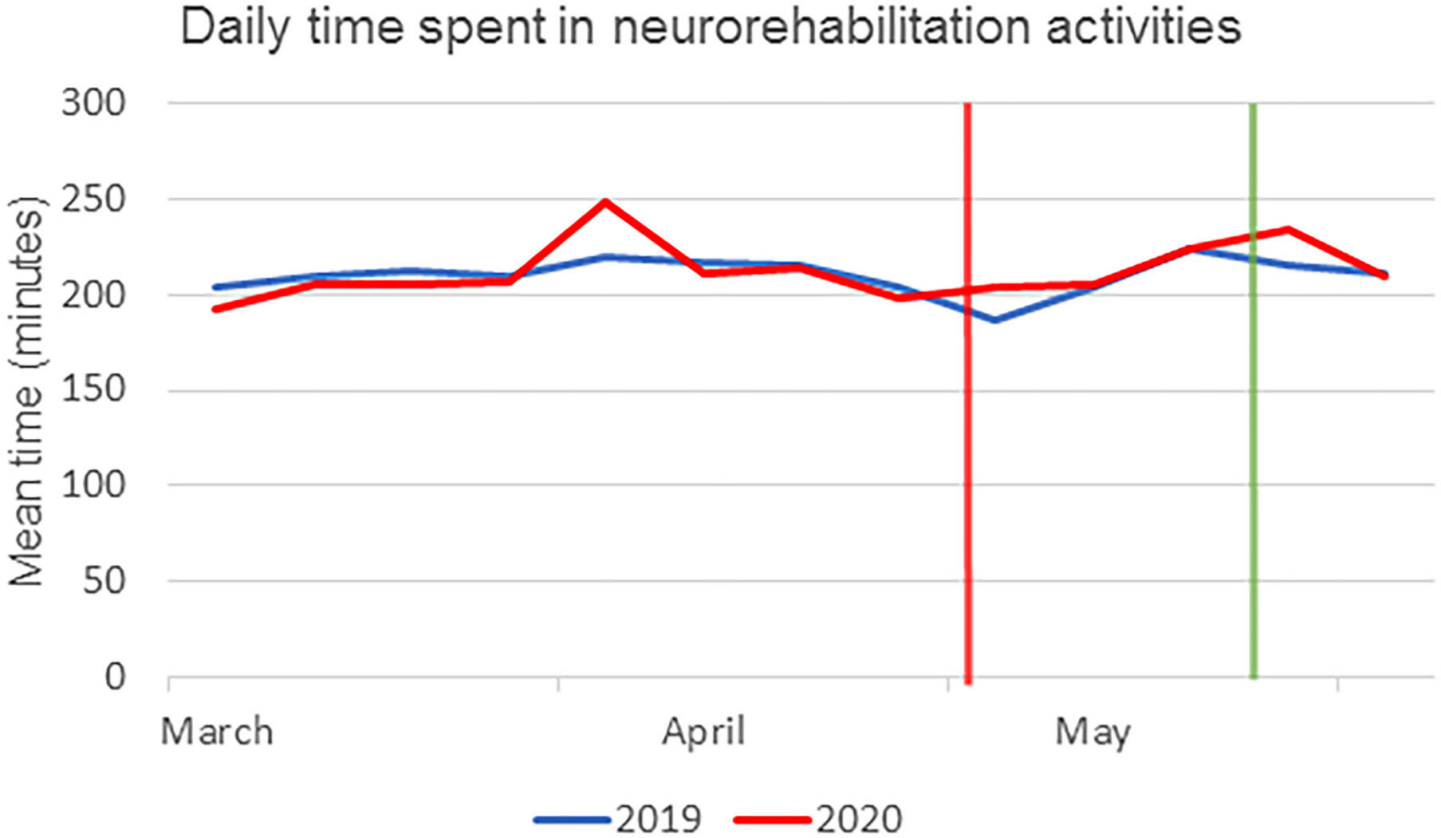
Mean time spent by patients in neurorehabilitation activities (in minutes), averaged by week in 2019 (blue line) and 2020 (red line). The vertical red line represents the day of the first positive case in our hospital, corresponding to the beginning of the temporary halt to new admissions. The green line represents the last day of this period of halted admissions.

The mean time spent by each patient in neurorehabilitation activities was not reduced in the period from March to May 2020 (global mean: 213 ± 15 min) with respect to the same period in 2019 (211 ± 10 min, *p* = 0.558), nor in the period after the first positive cases with the temporary halt to new admissions (211 ± 11 vs. 215 ± 10 min, *p* = 0.681). The number of patients discharged in the period from March to May 2020 was 71; their median BI-score was 69 (IQR = 50) with a treatment effectiveness of 48 ± 40% and a median length of stay of 49 days (IQR = 42). In the same period in 2019, the number of discharged patients was much higher at 233, with a median BI-score of 54 (IQR = 67, *p* = 0.049), an effectiveness of 47 ± 36% (*p* = 0.464) and a median length of stay of 63 days (IQR = 52, *p* = 0.001), as shown in Figure 3.

## Discussion

The aim of the present observational study was to report on how COVID-19 infections were monitored and contained in a large sample of inpatients and workers, and how infection management might have affected neurorehabilitation activities. Our results highlight three important findings about the management of the COVID-19 outbreak in neurorehabilitation.

First of all, the safety protocol and continuous monitoring performed with nasal swabs allowed us to identify 7 cases in 1,207 screened people. All the positive cases were asymptomatic, and only the planned monitoring allowed us to discover the presence of positive cases in the hospital. The prevalence of COVID-19 on the screened population in our hospital (0.6%) was slightly higher than that of the Italian population at 0.2% (11), but this was due to the fact that our sample was related to a hospital population.

The second finding is related to the clusterization of positive cases, with 6 out of 7 coming from the same ward. The odds ratio related to the environmental factor was 64.8 vs. an odds ratio of 3.85 for the category of persons (patients vs. clinical staff). This effect was already well-known and at the basis of social distancing and isolation of infected patients (12). Furthermore, this odds ratio may suggest the need to isolate the different wards of a hospital, reducing possible contacts among patients and/or workers in different wards. The protocols related to internal transfers, inspired by the Decrees of the Italian President of the Council of Ministers together with the scientific literature (4, 10), adopted severe measures and allowed us to counteract the pandemic. Among the adjunctive measures, it is important to mention the requirement of two negative nasal swabs within the last 48 h before discharge from acute wards for being admitted in our hospital, even if this was not required by the World Health Organization guidelines.

Moreover, the prompt interdiction of visitor entry, although this rule enormously overwhelmed nursing staff and neurorehabilitation professionals regarding the management of daily activities and the psychological isolation of inpatients with severe neurological disabilities and common neuropsychological disorders. Another factor that may have contributed to containing the outbreak could be the architectural structure of our wards, with wide bedrooms having only two beds each and the availability of a wide gym in each ward. Before COVID-19, some authors (13) reported that the architecture of hospital facilities does not influence nosocomial infection rates, concluding that there is a lack of stringent evidence to link hospital design and construction with the prevention of nosocomial infection. However, further studies (14–16) support the opposite idea, i.e., that the design of the physical environment influences nosocomial infection rates. The safety protocols, the large rooms and the presence of a gym in each ward, together with the presence of six isolation rooms with negative pressure, could be the key factors that limited the outbreak of SARS-CoV-2 in our hospital.

Finally, the third main finding of our study was that, during the containment of the pandemic in our hospital, the admissions were significantly reduced due to the temporary halt on admissions, but the mean time spent by inpatients in rehabilitation was not reduced by the emergency. It allowed us to provide the same level of treatment effectiveness. Despite many difficulties in discharging patients from our neurorehabilitation hospital due to the problems with managing the chronic phase of neurological diseases, the length of stay was shorter than that in 2019, according to a general process of reduction of length of stay already occurring before pandemic. The main problem was the dramatic reduction in amount of care reserved to outpatients, as also observed elsewhere (8).

Our study has some limits, the main of which is that the present study is an observational study based only on one hospital, without a population-based analysis and with some populations (i.e., stroke patients) that could be overrepresented. However, our study is similar to a previous one analyzing rehabilitation of outpatients, although ours is more focused on inpatients. The presence of COVID-19, its management and the contemporary management of neurorehabilitation makes this study important for other similar hospitals.

In conclusion, the prevalence of COVID-19 infection in our inpatient neurorehabilitation hospital was effectively managed, affecting the number of admissions and the rehabilitation of outpatients, but not that of inpatients, who achieved a good level of independence in the activities of daily living, similar to that of hospitalized patients in the same period in 2019. Six out of seven positive cases of SARS-CoV-2 were recorded in the same ward, and wide screening allowed us to contain the infection by isolating and transferring positive professionals and patients, respectively. These results suggest the need for repetitive and systematic screening of patients and clinical staff in neurorehabilitation hospitals to prevent outbreaks and to maintain a high amount of effective neurorehabilitation for inpatients.

## Data Availability Statement

The original contributions presented in the study are included in the article/supplementary material, further inquiries can be directed to the corresponding author/s.

## Ethics Statement

The studies involving human participants were reviewed and approved by Fondazione Santa Lucia ethics committee. The patients/participants provided their written informed consent to participate in this study. Written informed consent was obtained from the individual(s) for the publication of any potentially identifiable images or data included in this article.

## Author Contributions

GM and MI wrote the first draft of this manuscript. MI and SP performed all the data analysis. CC, AS, and AR conceptualized and supervised the study. SP, MG, MT, UN, RF, and MM revised the manuscript and provided important contributions. MB was responsible of all the swab analysis. All authors contributed to the article and approved the submitted version.

## Conflict of Interest

The authors declare that the research was conducted in the absence of any commercial or financial relationships that could be construed as a potential conflict of interest.
